# The UL16 protein of HSV-1 promotes the metabolism of cell mitochondria by binding to ANT2 protein

**DOI:** 10.1038/s41598-021-93430-2

**Published:** 2021-07-07

**Authors:** Shiyu Li, Shuting Liu, Zhenning Dai, Qian Zhang, Yichao Xu, Youyu Chen, Zhenyou Jiang, Wenhua Huang, Hanxiao Sun

**Affiliations:** 1 Institute of Genomic Medicine, College of Pharmacy, Jinan University, Guangzhou, 510632 China; 2 Department of Anatomy, School of Basic Medical Sciences, Southern Medical University, Guangzhou, 510515 China; 3 Department of Stomatology, Guangdong Second Traditional Chinese Medicine Hospital, Guangzhou, 510095 China; 4 Department of Microbiology and Immunology, College of Basic Medicine and Public Hygiene, Jinan University, Guangzhou, 510632 China

**Keywords:** Immunochemistry, Viral host response

## Abstract

Long-term studies have shown that virus infection affects the energy metabolism of host cells, which mainly affects the function of mitochondria and leads to the hydrolysis of ATP in host cells, but it is not clear how virus infection participates in mitochondrial energy metabolism in host cells. In our study, HUVEC cells were infected with HSV-1, and the differentially expressed genes were obtained by microarray analysis and data analysis. The viral gene encoding protein UL16 was identified to interact with host protein ANT2 by immunoprecipitation and mass spectrometry. We also reported that UL16 transfection promoted oxidative phosphorylation of glucose and significantly increased intracellular ATP content. Furthermore, UL16 was transfected into the HUVEC cell model with mitochondrial dysfunction induced by d-Gal, and it was found that UL16 could restore the mitochondrial function of cells. It was first discovered that viral protein UL16 could enhance mitochondrial function in mammalian cells by promoting mitochondrial metabolism. This study provides a theoretical basis for the prevention and treatment of mitochondrial dysfunction or the pathological process related to mitochondrial dysfunction.

## Introduction

Herpes simplex virus 1 (HSV-1) is a ubiquitous alphaherpesvirus, capable of both productive and latent infections in the human host^1^. Similar to all viruses, HSV-1 relies on the metabolic network of the host cells to provide energy and macromolecular precursors to fuel viral replication. However, it has been reported that, rather than passively relied on basal host cell metabolic activity, HSV-1 actively redirected host cell metabolism^2^. Mitochondria participate in a variety of cellular metabolic processes, of which functions are regulated by extrinsic and intrinsic stimuli including viruses. Mitochondria generate ATP through three main metabolic pathways, namely β-oxidation, tricarboxylic (TCA) cycle, and oxidative phosphorylation. As for HSV-1, it activates glycolysis through the engagement of the enzyme 6-phosphofructo-1-kinase (PFK-1)^3,4^ and increases anapleurotic influx to TCA cycleviapyruvate carboxylase (PC), feeding pyrimidine biosynthesis^5^, although by a different mechanism than human cytomegalovirus (HCMV)^6^. Studies on changes in cell metabolism by viral infections have become important in the field of virology. However, there is little known about the molecular mechanisms. We sought to find a mitochondrial-specific viral protein in HSV-1 as a mitochondrial stimulator to provide a basis for improving the physiological and pathological processes associated with mitochondrial dysfunction.

Microbial infection can cause changes in the function of mitochondria in host cells^7–9^. Many virus infections take mitochondria as the main target^10, 11^. Some viral infections enhance mitochondrial metabolism and activate the tricarboxylic acid cycle to provide energy and intermediate metabolites as power and raw materials for virus replication and assembly^12^. Some viral infections can change the structure of cell mitochondria in a short time, cause mitochondria to swell or even dissolve and destroy, and activate endogenous mitochondrial signal pathways leading to cell necrosis and apoptosis^13,14^. Therefore, mitochondria play an important role in the occurrence and development of diseases after virus infection. At present, there are more and more studies on the damage of mitochondrial function caused by virus infection. The excessive expression of viral protein in the hepatitis C virus (HCV) can cause a change of mitochondrial membrane potential, inhibit oxidative phosphorylation and increase the production of ATP, which may be the reason for the conversion of energy to glycolysis^15^. In the process of apoptosis of SL-1 cells induced by baculovirus AfMNPV, the number of mitochondria increased at first, then the mitochondria swelled, the mitochondrial cristae decreased and began to show vacuoles gradually, until the late mitochondrial pyknosis, the volume decreased greatly, and the apoptotic protein Bax was also transferred from the cytoplasmic matrix to the mitochondrial membrane to induce cell apoptosis. Therefore, it is of great significance to reveal the pathogenic mechanism of the virus to the host by studying the morphology and function of cell mitochondria after virus infection.

The mitochondria fulfill several key functions within cellular metabolic and antiviral signaling pathways, involving their central role in ATP generation. Viruses, as intracellular parasites, require from their cellular host the building blocks for the generation of their viral progeny and the energy that drives viral replication and assembly. Interestingly, recent studies have revealed that some viral proteins locate to mitochondria and interact with mitochondrial proteins. Viral proteins of herpesvirus HSV-1 located in mitochondria are UL7^16^, UL16^17^, US3^18^, UL12.5^19^ have been reported in other studies. It is also known that HSV-1 affects the activity of electron transfer chains through the inhibition of electron transport between complexes II and III, which is mediated by US3^18^. UL12.5 protein targets mitochondria and degrades mitochondrial DNA by nuclease function early in viral infection^19^.

Adenine nucleotide transporter (ANT), with a molecular weight of about 30,000 Daltons, exists as a dimer and is encoded by three different but related nuclear genes. Its isoforms are similar in humans as ANT1, ANT2, and ANT3, in other mammalian species^20^. These monomers are tissue-specific and their expression is sensitive to the special physiological conditions of cells.

In this experiment, we screened out the viral target protein binding to mitochondria by differentially expressed mitochondrial proteins of HSV-1 infected cells, and observed the role of the viral protein in stimulating cellular energy metabolism. In addition, we observed the effect of selected viral proteins on mitochondrial function in the model of d-Gal-induced mitochondrial dysfunction.

## Materials and methods

### Viruses and cells

The virus strain HSV-1 and human umbilical vein endothelial cells (HUVEC) was preserved and cultured in our laboratory^21^. HUVEC cells were inoculated in six-well plates at the density of 9 × 10^4^ cells/mL and cultured overnight in an incubator containing 5% CO_2_ at 37 ℃. Wash with phosphate buffer solution for 3 times to remove the culture medium. In this study, 55 mmol/L (10 g/L) d-Gal was selected to act on HUVEC cells to establish a fine cell senescence model, and the related changes of mitochondria during senescence were analyzed. HUVEC cells were inoculated with high glucose (25 mmol/L d-glucose) DMEM medium containing 10% fetal bovine serum and 1% penicillin–streptomycin solution. HUVEC cells were cultured at 37 °C 5% CO_2_ for 24 h. after the cells were attached, the cells were cultured in 55 mmol/L d-Gal medium for 72 h.

The cell experiment was divided into three groups: normal control group, d-Gal group, d-Gal transfection UL16 group.

### Construction of UL16 plasmid and siRNA vector

The UL16 gene of HSV-1 was cloned into pcDNA3.1 plasmid (Invitrogen, Carlsbad, CA) to construct pcDNA3.1-UL16-GFP plasmid. The mutant vector pcDNA3.1-UL16-(C357S) was constructed by creating a mutation of the 357th amino acid from cysteine to serine. The siRNA vector of UL16 was constructed by pSINsi-mU6 DNA. All expression plasmids were verified by sequencing^22^. The oligonucleotide UL16 of primers was used for (5′-CCGAATTCAAGCTTATG CTCCGCAACGAGCC) and UL16-rev (5′-CGGGATCCTTTATTGAAAAATATCAA).

### Microarray analysis

HUVEC cells grown on cover slips were infected with HSV-1 (F) at an MOI of 5. After 24 h, cells were lysed, total RNA was isolated with Trizol (Invitrogen, Carlsbad, CA) and purified with RNeasy Mini kit (Qiagen, Valencia, CA). Three biological replicates were performed for each experimental condition. Samples preparation and hybridization to Whole Human Genome Gene Expression Microarray were performed at Kangcheng Bio-tech (Shanghai, China). Use DESeq software to perform differential gene expression analysis on cell samples, mark *p* value < 0.01 and differential expression multiple >  = 2 (|log2|> = 1) as differentially expressed genes, FDR threshold < 0.05. Functional analyses of gene selected were carried out using Gene Ontology project (http://www.geneontology.org). Genes with similar expressed tendency were classified by cluster analysis based on values of normalized expression (correlation > 0.99). Endeavour software (http://homes.esat.kuleuven.be/~bioiuser/endeavour/endeavourweb.php) and ToppGene (http://toppgene.cchmc.org) ^23,24^ were used for prioritization based on the similarity of the known genes.

### Quantitative real time PCR

To validate microarray results, mRNA expression was monitored using quantitative real time PCR (qRT-PCR) for the selected genes with β-actin expression used to normalize for total RNA content. The genes were amplified using respective primer as described in Supplementary Information Table S1. PCR products were separated on 2% agarose and stained with ethidium bromide. What’s more, the mRNA expression of seven genes also by qRT-PCR, which was performed with the Time PCR Detection system (Bio-Rad Laboratories, Inc, USA) and the Maxima SYBR Green qPCR Master Mix (2x) (Fermentas, Germany).

### Mitochondrial protein isolation

Mitochondria were isolated from HSV-1 infected cells and uninfected control cells, using mitochondrial isolation kit (Thermo 89, 801, USA). The cells were suspended in 800 μL of LBSA / Reagent A solution at 4 ℃ and incubated on ice for 2 min. 10 μL of mitochondrial separation reagent B was added to the cell suspension and incubated on ice for 5 min. Then, 800 μL of mitochondrial separation reagent C was added to the tube and mixed. Finally, the mitochondria were separated after centrifugation, washed twice with 500 μL of wash buffer and stored at − 80 °C until use.

Mitochondrial protein sample preparation, mitochondria suspension was added to DIGE-specific lysis buffer (7 M urea, 2 M thiourea, 30 mM Tris–HCl, 4% CHAPS, at pH 8.5), incubation on ice for 30 min, then centrifuge (20,000 × *g*) at 4 ℃. The supernatant was centrifuged at 15,000 × *g* for 30 min at 4 ℃ to remove salts and other impurities, and then resuspended in DIGE-specific lysis buffer. The protein solution was collected and the total protein concentration was measured using a BCA Protein Assay Kit (Thermo Fisher Scientific, USA).

### Western blot analysis of mitochondrial proteins

The mitochondrial protein sample was mixed with the loading buffer and heated at 100 ℃ for 8 min, then separated by SDS-PAGE (Tris-HEPES-SDS gradient 4 to 20% gels) and transferred to a PVDF membrane. The membrane containing the transferred protein was blocked with 5% skim milk (150 mM NaCl, 10 mM Tris, 0.1% Tween-20, pH 8.0) in TBST. The blocked membrane was incubated with anti-β-actin (1:1000, Santa Cruz, CA, sc-47778), anti-histone 3 (1:1000, Abcam, ab1791), anti-VDAC1 (1:2000, Abcam, ab154856), anti-caspase-3 (1:1000, CST, 9665S), anti-cleaved caspase-3 (1:1000, CST, 9664L), anti-PURA (1:1000, Abcam, ab84928), anti-PSPC1 (1:1000, Abcam, ab184123), anti-Dynamin-1 (1:2000, Abcam, ab40758), anti-SYN2 (1:5000, Abcam, ab76494), anti-cytochrome c oxidase subunit (1:2000, Abcam, ab150422), anti-Complex I (1:1000, Abcam, ab110245), anti-beta tubulind (1:1000, Abcam, ab6046) in TBST buffer 4 ℃ overnight. After washing in TBST, membranes were incubated with a 1:3000 dilution of anti-rabbit or anti-mouse IgG HRP secondary antibody diluted in TBST for 1 h. Subsequently, the membrane was washed in TBST (4 × 10 min) and developed using a chemiluminescent reagent from an ECL kit (Thermo Scientific Pierce ECL, USA). Blots were detected on a phosphor imager and analyzed using ImageQuant 350 software.

### MALDI-TOF–MS mass spectrometry

The position and molecular weight of the bands in the two gels were compared and grayscale analysis was carried out with Image J software (http://imagej.nih.gov/ij/plugins/). The samples with statistically significant differences (*p* < 0.05) were selected for MS mass spectrometry analysis. SDS-PAGE electrophoresis was performed with 1.5 mg protein sample according to the above western blot steps. The gel was stained with Coomassie brilliant blue solution (containing 0.12% Coomassie Brilliant Blue G-250, 20% ethanol, 10% phosphoric acid, 10% ammonium sulfate). After stainning, manually remove the band of protein ladder from the gel. Each gel was digested with trypsin (Promega Corp, WI, USA) overnight at 37 ℃, for MALDI-TOF–MS mass spectrometry.

MALDI-TOF/TOF mass spectrometer (AB SCIEX 5800, USA) was used for protein identification, 0.6 μL of protein sample was treated with 1 μL of 10 mg/ml α-cyano-4-hydroxycinnamic acid (CHCA) at 0.1% TFA, 50% acetonitrile (ACN), directly crystallized on the target and dried at room temperature for detection. The equipment is set in linear mode, ion acceleration voltage 20 kV, N2 laser wavelength 337 mm, pulse width 3 ns, ion delay extraction 500 ns, vacuum 5 × 10^5^ Pa, the spectrum is externally calibrated.

### Coimmunoprecipitation and immunoblotting

Coimmunoprecipitaion and immunoblotting were performed as described previously^16^. Briefly, HUVEC cells, in 60 mm dishes were transfected with pCMV-UL16-Flag. At 16 h post-transfection, cells were harvested, washed with PBS, and lysed in 500 μL NP40 buffer (50 mM Tris–HCl pH 8.0), 120 mM NaCl, 0.5% NP40, 1 mM PMSF). The supernatants obtained after centrifugation were precleared by incubation with protein G-Sepharose beads for 30 min at 4 ℃. After a brief centrifugation, the supernatants were reacted with the anti-UL16 rabbit polyclonal antibody for 2 h at 4 ℃. Protein G sepharose beads were then added and allowed to react with rotation for an additional 1 h at 4 ℃. The immunoprecipitates were collected by a brief centrifugation, washed extensively with NP40 buffer, and analyzed by immunoblotting with anti-Flag monoclonal antibody. In other experiments, HUVEC cells were transfected with pFLAG-CMV-2 or pCMV(f)ANT2 as described above. At 24 h post-transfection, transfected cells were infected with HSV-1 at an MOI of 5. At 24 h after infection, the cells were harvested and subjected to immunoprecipitation with the ANT2 antibody and immunoblotting with the anti-Flag antibody as described above.

### Glycolytic flow analysis and metabolome analysis of HUVEC cells infected UL16

When the infection group cells have obvious cytopathic effect, extracted the metabolites of culture medium respectively. The extract is analyzed by means of 1 H NMR. To evaluate whether UL16 could affect aerobic oxidation of glucose, HUVEC cells in 6-well plates were transfected with expression plasmids for UL16. After transfected 24 h, supernatants of mock- (without UL16) and UL16-transfected cells were collected at 4, 8, 16 and 24 h. In addition, the concentrations of glucose, pyruvate and lactate were measured using Glucose Assay Kit, Pyruvate Assay Kit and Lactate Assay Kit, respectively (Biovision, Milpitas, CA). Calculation of specific rates of glucose uptake was performed as described previously^25^. The levels of ATP in our cells were measured by the luciferin-luciferase method^26^, HUVEC cells were cultured in a six-well plate (3 × 10^5^ cells per well) and lysed at 16 h p.i. The enzyme activity of 6-phosphofructo-1-kinase, pyruvate kinase and lactate dehydrogenase were measured by the corresponding enzyme activity detection kit (Sigma- Aldrich, St. Louis, USA).

### Measurement of mitochondrial membrane potential

For measurement of mitochondrial membrane potential (MMP), cells cultured at low density on 24 well glass-bottom plate were incubated for 20 min at 37 ℃ with the following probes: 40 nM tetramethyl rhodamine methyl ester (TMRM, Molecular Probes, Invitrogen, Carlsbad, CA, USA), 1 μM Mito Tracker Green (MTG, Molecular Probes) and 2 μM Hoechst 34,580 (Molecular Probes) to monitor MMP. The fluorescence value was detected at wavelength by enzyme labeling instrument^27^.

### Measurement of mitochondrial ROS

For cells selected from the UL16 group and the d-Gal group, each sample was washed with PBS, and then a PBS solution containing 5 μmol/L DCFH-DA was added, mixed well, and incubated at 37 °C for 30 min. Wash twice with PBS buffer to remove probes that have not entered the cells, and incubate at 37 °C for 30 min. Centrifuge at 8000 rpm for 2 min, and resuspend the cells in 1 ml PBS buffer. Detect the fluorescence intensity of each tube with a flow cytometer (excitation wavelength is 488 nm, emission wavelength is 530 nm), count 10,000 cells, and find the average fluorescence intensity. Observe after excitation with ultraviolet light (excitation wavelength 380 nm, absorption wavelength 420 nm) under a fluorescence microscope.

### Confocal microscopy

HUVEC and hADSCs were seeded on cover slips in 12-well dishes prior to transfection. Transfections were performed using Lipofectamine 2000 (Invitrogen, Carlsbad, CA) according to the guidelines specified by the manufacturer. Cells were incubated 2 mL DMEM medium containing 5 μg/ml Hoechst 33,342 at 37 ℃ for 100 min, the transfected cells were washed with cold PBS and then incubated with medium containing 100 nM MitoTracker Green (Invitrogen, Carlsbad, CA) at 37 ℃. Which stains mitochondria in live cells, and fixed in 3% paraformaldehyde. The number of mitochondria in cell after differentiation were detected by flow cytometry.

### Statistical analysis

All data are expressed as arithmetic mean ± standard deviation (SD, n ≥ 3). Data were processed with GraphPad Prism 5.0, using one-way ANOVA, where appropriate. *p* < 0.05 was considered statistically significant.

## Results

### Microarray data analysis to identify differentially expressed genes after virus-transfected cells

**Figure 1:**
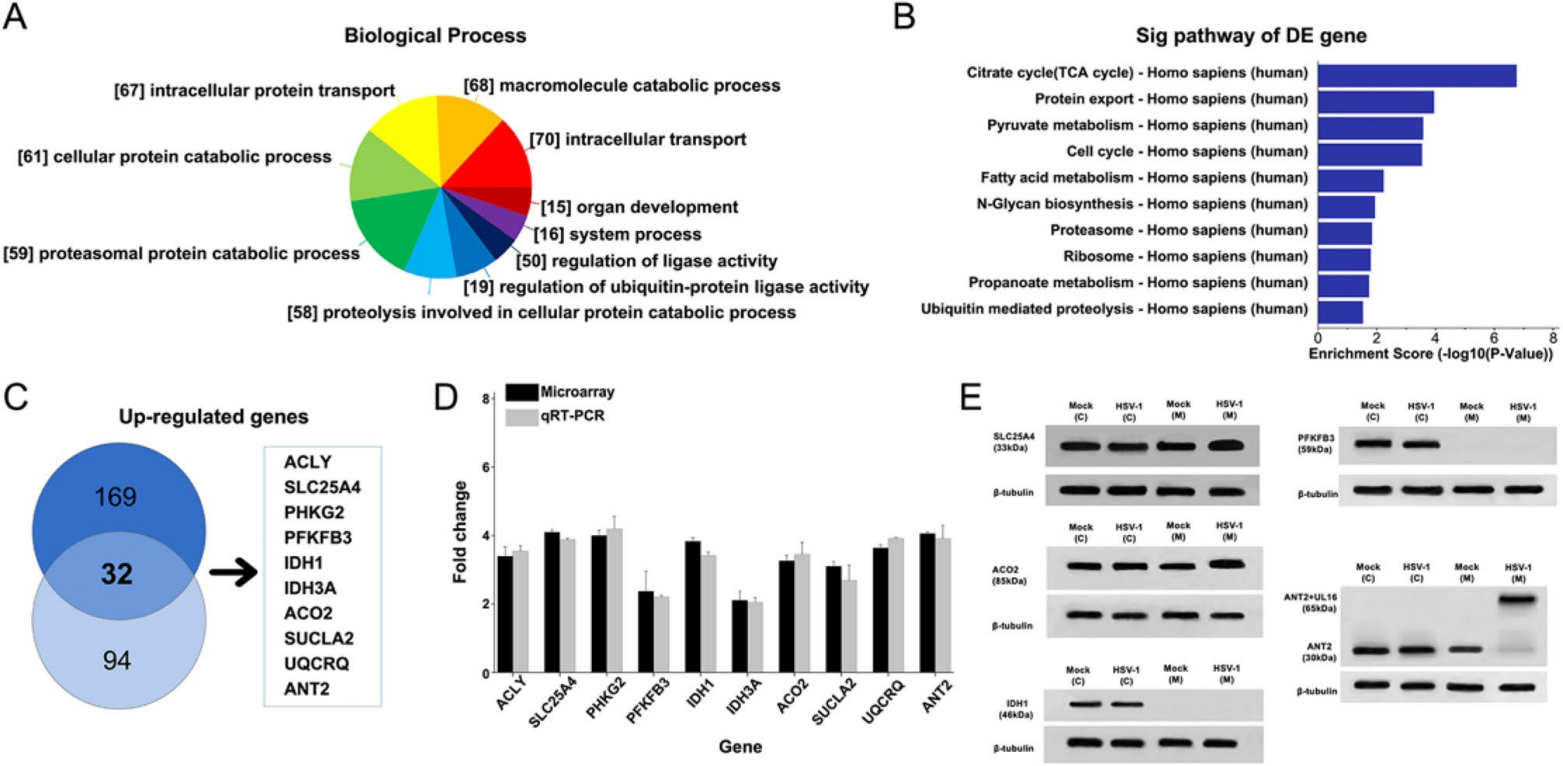
Microarray data analysis and validation of array data by quantitative real-time PCR. HUVEC cells were infected with HSV-1 at an MOI of 5. After 24 h post-infection, compared to Mock cells, the HSV-1 mutant infected cells revealed the differentially expressed genes, especially upregulated genes, were classified into different functional categories according to Gene Ontology annotation. DESeq 3.4.4 (https://bioconductor.org/packages/release/bioc/html/DESeq.html), Endeavour 3.71 (http://homes.esat.kuleuven.be/~bioiuser/endeavour/endeavourweb.php), ToppGene (http://toppgene.cchmc.org). (**A**) Upregulated genes were described by biological process. (**B**) The differentially expressed genes were also analysed by KEGG Pathway. The top ten enrichment scores of upregulated pathways in HSV-1 infected cells. (**C**) Through the screening of differentially expressed genes in multiples of 2 times or more than 2 times, Venn diagram of the significantly upregulated genes in HSV-1 infected cells compared with Mock cells. (**D**) The qRT-PCR data were normalized to β-actin expression. Fold changes were determined by calculating the ratio of the mean expression values from the control and samples. Each value represents the mean of triplicates and barsindicate standard deviation. (**E**) Western blot of HSV-1 infected cells at 24 h, the differentially expressed proteins in cytoplasm and mitochondria. Mock as control group (empty plasmid transfection) (ImageQuant 350 software).

To identify cellular genes whose expression level change during HSV-1 infection, HUVEC cells were infected with HSV-1 which had been successfully constructed. We grouped cellular genes according to the GeneOntology annotation of biological progress. The majority of genes fell into functional categories that were known or likely to be important in host responses to viral infections, including intracellular protein transport, macromolecule catabolic process, proteasomal protein catabolic process (Fig. 1A). In this GeneOntology annotation of cellular component and molecular function, most upregulated genes belonged to the categories of response to mitochondrion, organelle membrane, organelle lumen (Fig. 1B) and ATP binding, signal sequence binding (Fig. 1C), respectively. What's more, most differentially expressed genes mainly affected TCA cycle, protein export, pyruvate metabolism (Fig. 1D). We concluded that the majority of differentially expressed genes were involved in cellular energy yielding metabolic pathways.

Most of the known upregulated genes may possess more interactions with other proteins. By the network-based analysis of topological features, we identified 169 genes (specificity = 80%) and 94 genes (sensitivity = 90%) from all the upregulated genes which are obviously affected. Then we combined the two gene lists and obtained a total of 32 overlapped genes due to the variability. Endeavour and ToppGene of the functional consistency analysis could identify 85% and 91% of known associated genes, respectively. Through the screening of differentially expressed genes in multiples of 2 times or more than 2 times. Then we selected ten genes by each of the two methods (Fig. 1C). Including ATP citrate lyase (ACLY), acute monocytic leukemia tumor associated antigen (solute carrier family 25 member 4, SLC25A4), phosphorylase kinase gamma 2 (PHKG2), phosphofructo-2-kinase/fructose-2,6-bisphosphatases 3 isozyme (PFKFB3), Adenine nucleotide transporter 2 (ANT2), Isocitrate Dehydrogenase NADP^+^ (IDH1), Aconitate 2 (ACO2), Succinate-CoA Ligase ADP-Forming Beta Subunit (SUCLA2), Isocitrate Dehydrogenase 3 NAD + Alpha (IDH3A), Ubiquinol-Cytochrome C Reductase (UQCRQ). We screened candidate target genes for HSV-1 affecting host cell metabolism. Subsequently, molecular annotation of known data is usually performed on 10 differentially dominant candidate target genes to improve accuracy (Supplementary Information Table S2).

### Confirmation of microarray data for differentially expressed genes by qRT-PCR

To evaluate the reliability of the expression changes detected by the microarray analysis, we used qRT-PCR to analyze ten selected genes (the entire range of expression changes from 2.5- to 4.1-fold, *p* < 0.05). The β-actin transcript was chosen as an internal control since expression of this message was not affected by HSV (Fig. 1D). The results demonstrated that the detected genes were in accordance with microarray data, merely with a very trivial difference.

The differentially expressed genes on the cytoplasm and nucleus are IDH1, PFKFB3 (Fig. 1E), PHKG2, ACLY (Supplementary information Fig. S1).

### UL16 expression after transfection and ATP level after UL16 combined with ANT2

We performed gel extraction and digestion of the molecular bands bound by ANT2 and viral proteins, and analyzed the molecular weight of the peptides from the digestion of the bound proteins by MALDI-TOF–MS and searched the WSSV ORF database (Supplementary information Fig. S2). Amino acid sequence of UL16 protein were C-P–C-V-A-P–C-L-W-A-K-M. The target red peptide segment is the amino acid sequence of the UL16 protein. MS analysis revealed that the peptide mass matched the signature peptide of the UL16 protein within 100 ppm.

**Figure 2:**
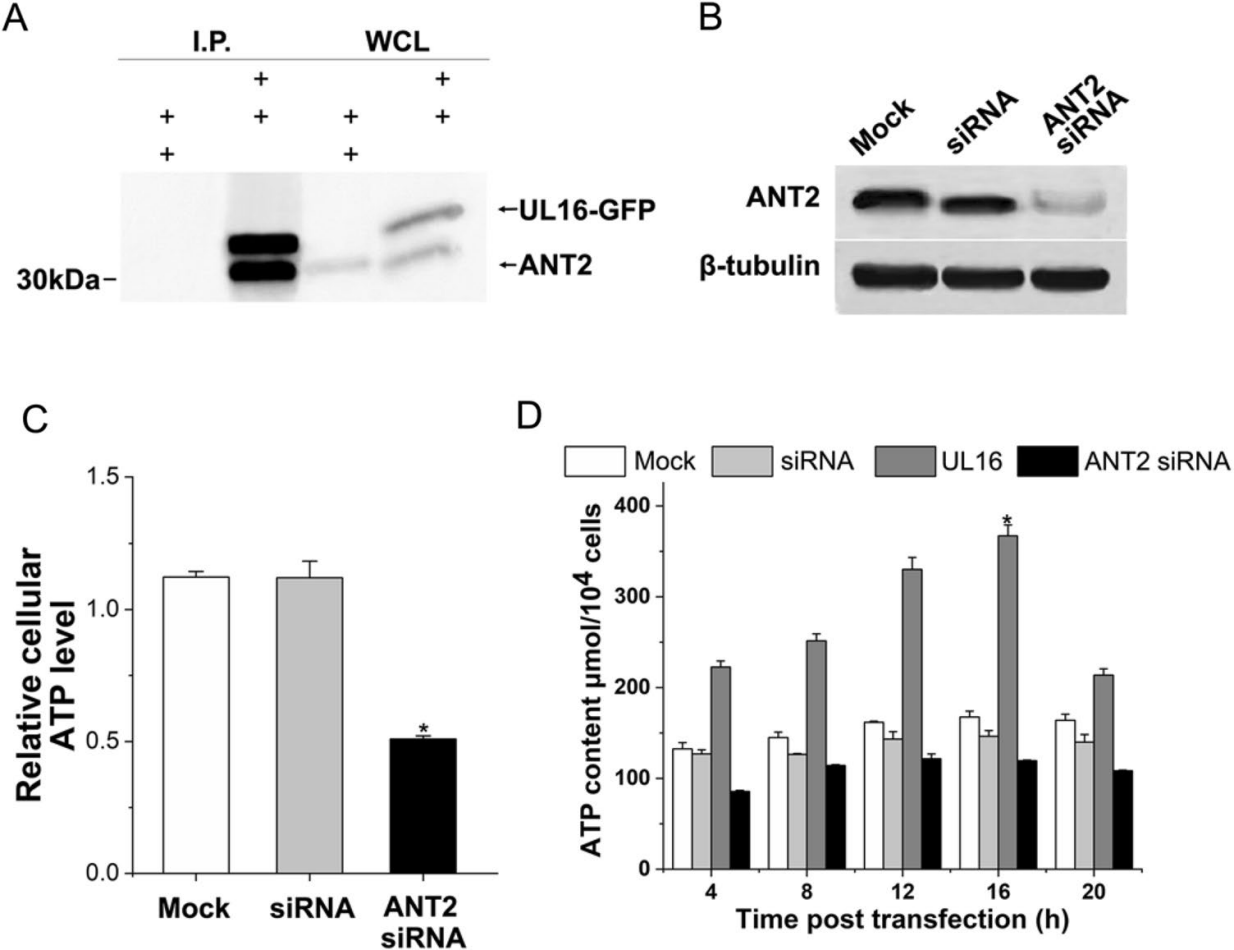
UL16 protein binding to ANT2. (**A**) Coimmunoprecipitation assay demonstrates that Flag-UL16 and ANT2 coexpression in HUVEC cells (Image J1 software http://imagej.nih.gov/ij/plugins/). (**B**, **C**) Immunoblots and cellular ATP levels of ANT2 at 24 h, after pSINsi-UL16 transfection. (**D**) ATP levels in 20 h of HUVEC cells, between mock cell, siRNA (control siRNA) transfection group, UL16 transfection group, and ANT2 siRNAs transfection group. The data shown in each panel are representative of three independent experiments.

To clarify the function(s) of UL16 in host cell metabolism, we tried to identify the host cellular proteins that interact with the UL16 protein. Six differential expressed genes (including Slc25A4, SUCLA2, UQCRQ, ANT2, IDH3A and ACO2) located in mitochondria, were seletced to verify whether UL16 in fact associates with these genes. The results no combination except ANT2 (Fig. 2A). UL16 was able to couple with ANT2 in HUVEC cells.

HUVEC cells transfected with siRNA vector of were solubilized and immunoprecipitated with the anti-UL16 polyclonal antibody. The immunoprecipitates were then subjected to immunoblotting with the anti-Flag antibody. As shown in Figure, the UL16 antibody coprecipitated UL16 with Flag epitopetagged ANT2 when UL16 and ANT2 were coexpressed in HUVEC cells (Fig. 2B). By using a modification of the gel overlay assay. With knowledge of the presence of ANT2 in mitochondria, cell lysates were assayed and a protein of 30 kDa was found. We further investigated the effect of UL16 on cellular ATP levels. HUVEC cells transfected with the siRNA expression vector of ANT2 showed that the ATP level of cells was lower than that of the empty plasmid transfection group and the scrambled siRNA transfection group 24 h after transfection (Fig. 2C). Compare the ATP levels of HUVEC cells in the mock group, siRNA transfection group, UL16 transfection group, and ANT2 siRNAs transfection group within 20 h. At 4, 8, 12, 16 and 20 h after transfection, the ATP level was significantly improved and continued to increase, and decreased after reaching the highest at 16 h (Fig. 2D).

### Effect of UL16 transfection into HUVEC on mitochondrial aerobic oxidation

The previously identified differentially expressed proteins are closely related to mitochondria, we try to find out the relationship between mitochondrial specific viral protein and energy metabolism. we examined the mitochondrial functions in UL16 transfected cells.

**Figure 3:**
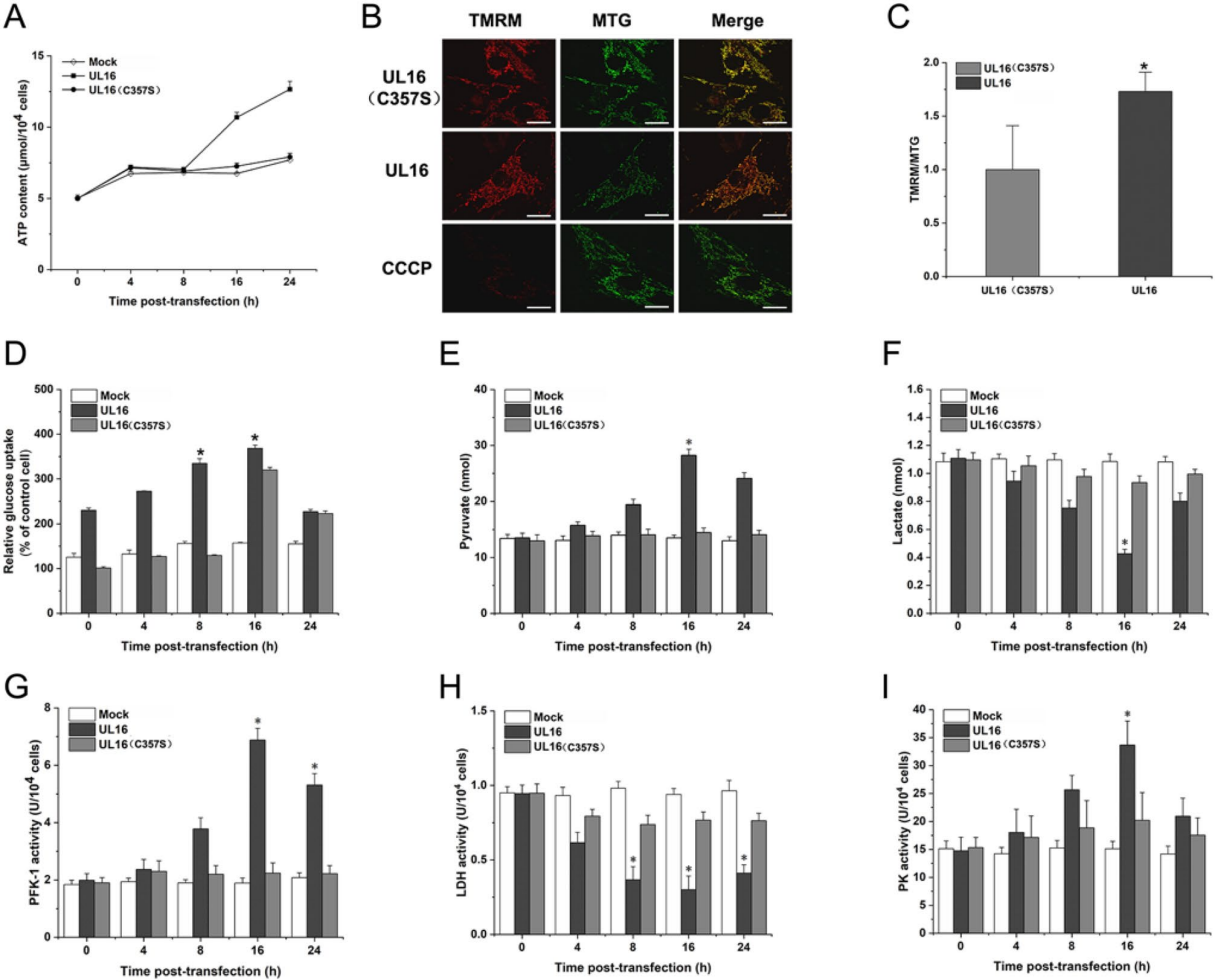
Test for aerobic oxidation in HUVEC after UL16 transfection. (**A**) HUVEC cells was transfected with UL16, UL16 (C357S) and MOCK (empty plasmid), respectively. After transfection, HUVEC cells transfected with or without virus protein at moi0.5 were cultured for 24 h and then subjected to the measurement of ATP content. (**B**, **C**) Comparison of MMP between mock cells (upper) and UL16 transfected cells (lower). Data are the means ± SD of values from three fields containing more than 100 cells (bar.10 μm, *p < 0.05, **p < 0.01). (**D**) HUVEC cells were transfected with UL16, UL16 (C357S), mock (empty plasmid). At different times post-transfection, the supernatants were collected, and glucose consumption was measured. (**E** and **F**) The concentrations of pyruvate, and lactate were measured at different time points after transfected. (**G**, **H** and **I**) The monolayers from mock-, UL16 and UL16 (C357S) transfected HUVEC cells were cultured for 24 h and then PFK-1, LDH and PK activity were measured. These enzyme activities were measured by the corresponding enzyme activity detection kit at different time points after transfected. Each value represents the mean of triplicates and barsindicate standard deviation. *Represents a *p* value below 0.05, **represents a *p* value below 0.01 for comparisons between mock- and UL16, UL16 (C357S) transfected cells.

We found that the content of ATP in UL16 group was higher than that in mock group and UL16 (C357S) group, and the mitochondrial function was significantly increased (Fig. 3A). We also detected the mitochondrial membrane potential (MMP) of UL16 transfected cells, MMP represents an important parameters of cell metabolism and mitochondrial energy status, which reflects to the mitochondrial electron transport activity that promotes ATP production in electron transport chain. Therefore, we performed the experiments to assess the status of MMP during UL16 transfection at moi5 for 24 h. On the other hand, HUVEC cells displayed higher red: green fluorescence intensity compared to non-infected cells, which indicated that persistent infection with UL16 caused a sustained elevation in MMP (Fig. 3B, C).

### UL16 transfection into HUVEC promotes aerobic oxidation of glucose

Glycolysis and the TCA cycle form the backbone of central carbon metabolism in mammalian cells. Therefore, we decided to further investigate the effect of UL16 protein on the aerobic oxidation of glucose. We found that glucose uptake in HUVEC cells transfected with UL16 seemed to increase at 8 h after transfection, but more significantly at 16 h after transfection, which was measured by the decrease of glucose concentration in the supernatant of HUVEC cells (resulting in a more reasonable statistical significance; *p* < 0.01) (Fig. 3D).

The key rate-limiting regulatory enzymes of the glycolytic pathway are PFK-1 and pyruvate kinase (PK). Thus, we next investigated whether UL16 regulated those key rate-limiting enzymes of aerobic oxidation of glucose. We found that the concentration of pyruvate increased significantly and the concentration of lactic acid decreased 16 h after UL16 transfection, but changed little in mock group and UL16 (C357S) group (Fig. 3E, F). PFK-1 and competitive activity increased 16 h after transfection of UL16, slightly increased in UL16 mutant group, and almost no change in mock group (Fig. 3G–I). In parallel, we also analyzed other key-regulator of aerobic oxidation of glucose and found lactate dehydrogenase (LDH) was influenced by UL16 transfection, which suggested that UL16 remarkably decreased LDH activity and at 16 h post-transfection but only slightly decreased in cells transfected with mock. In conclusion, those results could be explained by enhanced glycolytic catabolic flux, leading to increasing the intracellular ATP content.

### UL16 transfection into HUVEC promotes the process of oxidative phosphorylation

**Figure 4:**
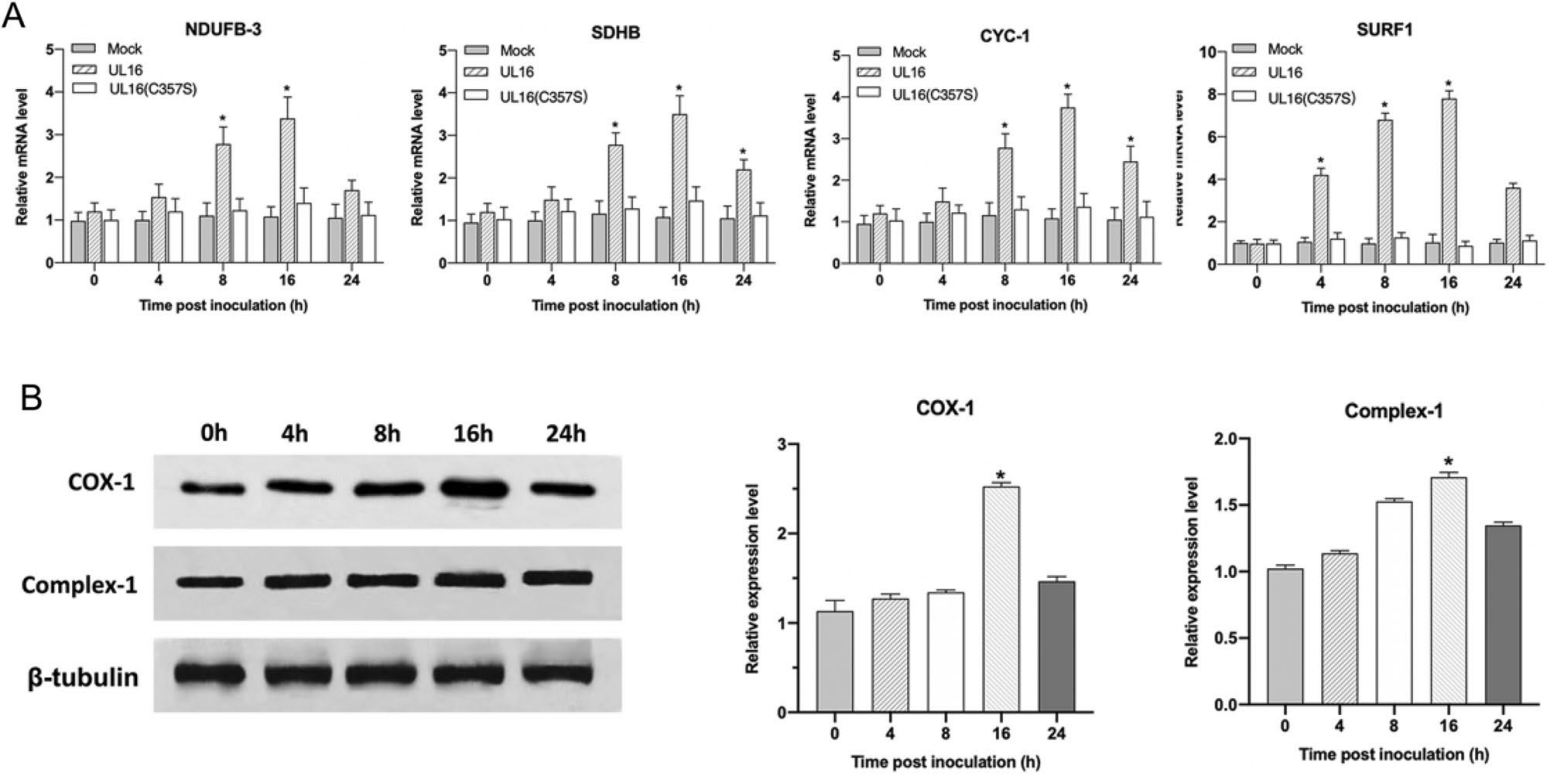
Text for the mRNA abundance of several Oxphos-related enzymes. (**A**) The mRNA expression of NDUFB3, SDHB, CYC1 and SURF1 were quantified by qRT-PCR technique as described in Materials and Methods. (**B**) Additionally, cells were lysed at indicated times and immunoblotted using anti-Complex I, COX I, and β-tubulin antibodies. Each value represents the mean of triplicates and barsindicate standard deviation. *represents a *p* value below 0.05 for comparisons between mock, UL16 (C357S) and UL16-transfected cells.

Oxidative phosphorylation (OXPHOS) is the metabolic pathway in which the mitochondria in cells use their structure, enzymes, and energy released by the oxidation of nutrients to reform ATP. To further analyze UL16 protein affected the intracellular energy system, we tested the activity of electron transport chains in mitochondria. NADH dehydrogenase (ubiquinone) 1 beta subcomplex subunit 3 (NDUFB3). Succinate dehydrogenase subunit B (SDHB), Cytochrome c-1 (CYC1) and Surfeit locus protein 1 (SURF1) were subunits of four membrane-bound complexes, including Complex I, II, III and IV. Therefore, we detected the mRNA expression of four genes by qRT-PCR technique, and the results showed that the level of UL16 mRNA increased significantly at 16 h after transfection. Sixteen hours after UL16 (C357S) transfection, the level of mRNA increased slightly, but there was almost no change in the mock group (Fig. 4A). Moreover, Cytochrome c oxidase subunit I (COX I) was one of three mitochondrial DNA (mtDNA) encoded subunits of respiratory complex IV. We further analyzed the protein expression of Complex I and COX I by western blotting. In truth, an increase in the protein expression starting at 4 h post-transfection and more pronounced expression of Complex I and COX I all at 16 h were observed (Fig. 4B). Overall, UL16 protein could activate electron transport chains activities through the protein expression increasing, such as Complex I, II, III and IV.

### Effect of UL16 transfected HUVEC treated with d-Gal on mitochondrial metabolism

**Figure 5:**
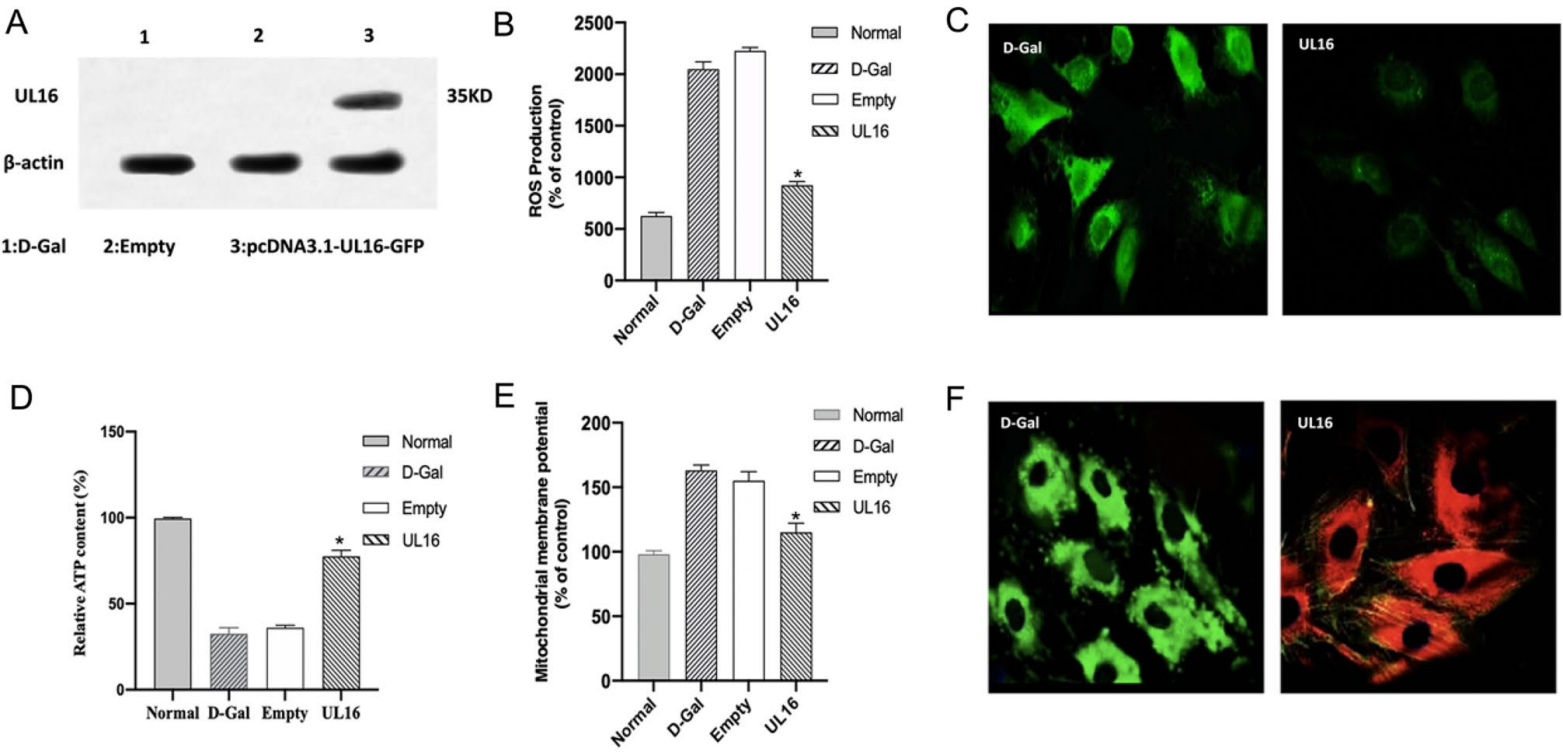
The mitochondrial function test of the mitochondrial damage model induced by d-Gal after UL16 transfection. (**A**) It was verified that UL16 had been successfully transfected into the HUVEC cells induced by d-Gal. (**B**, **C**) The intracellular ROS level was detected in d-Gal group and UL16 group. (bar.50 μm, *p < 0.05, **p < 0.01). (**D**) UL16 was transfected into HUVEC cells induced by d-Gal, and then the content of ATP was measured in UL16 group, d-Gal group, Noraml group and Empty group. (**E**, **F**) The mitochondrial membrane potential of cells in d-Gal group and UL16 group was measured. electron microscope. (bar.20 μm, *p < 0.05, **p < 0.01).

d-Gal has been widely used to establish aging animal models. In vitro studies, it is mainly used to evaluate the mitochondrial toxicity and mitochondrial oxidation ability of drugs. In this paper, we selected 55 mmol/L d-Gal to act on HUVEC cells to establish the model of mitochondrial dysfunction. Through the Flag tag carried on the UL16 gene, we carried out Western bolt analysis on the transfected HUVEC cells, and found that compared with the transfected empty plasmid, we found a band in 35 KD, which was consistent with the theoretical value of UL16 protein (Fig. 5A), which indicated that UL16 was successfully transfected into the cell and could be stably expressed in the cell.

Mitochondrial dysfunction can lead to an increase in the level of intracellular ROS. The increase of intracellular ROS level induced by d-Gal could be inhibited by UL16. The level of ROS in UL16 group was significantly lower than that in d-Gal group (Fig. 5B, C), indicating that UL16 could significantly reduce the level of oxidative stress in HUVEC cells. Induced by d-Gal, the function of mitochondria was impaired and the content of ATP in cells decreased significantly. We first detected the content of intracellular ATP after successful transfection of UL16, and found that UL16 could significantly increase the level of intracellular ATP, indicating that UL16 could enhance the level of intracellular oxidative phosphorylation and improve the function of mitochondria (Fig. 5D). In addition, the most intuitive manifestation of mitochondrial dysfunction is the decrease of mitochondrial membrane potential. We used JC-1 to detect the changes of mitochondrial membrane potential, showing green fluorescence when the mitochondrial membrane potential was low and red fluorescence when the mitochondrial membrane potential was maintained at a high level. The results showed that HUVEC cells showed green fluorescence induced by d-Gal, indicating that the mitochondrial membrane potential was at a low level at this time. When the cells were transfected with UL16, the cells showed red fluorescence, which indicated that the transfection of UL16 increased the mitochondrial membrane potential and saved the decrease of mitochondrial membrane potential induced by d-Gal (Fig. 5E, F).

## Discussion

Studies on the metabolic changes of virus-infected cells have become important in the field of virology^2, 5, 28^, we have screened out several differentially expressed genes that affect metabolism after HSV-1 infection by gene chip technology. Then we identified the viral protein UL16 binds to the differentially expressed gene ANT2. Although previous studies showed that UL16 was found to contain specific sites located on the mitochondria^17^, the mechanism of interaction with mitochondria, and its host proteins were not found^16^. However, our study has demonstrated for the first time that UL16 protein can co-precipitate with ANT2 in host cells. This reveals a new molecular mechanism by which HSV-1 affects host cell energy metabolism. Adenine nucleotide transporter (ANT) is the most important signal transduction protein in mitochondrial intima, which promotes the transport of ADP/ATP between cytoplasm and mitochondrial matrix. There are three isomers of ANT1, ANT2 and ANT3 in ANT. In the study of yeast kinetics, it was found that ANT2 was more involved in energy supply, and the transport efficiency of ANT2 to ATP/ADP was much higher than that of ANT1^29–31^. Our study shows that HSV-1 UL16 protein can target ANT2 protein on the mitochondrial membrane of host cells, activate the cellular energy metabolism pathway, and significantly increase the content of intracellular ATP. The UL16 protein activates the cell's energy system, producing more ATP, in the mitochondria and then activating the activity of ANT2, which accelerates the transport of ATP from the mitochondria to the cytoplasm and also accelerates the transport of ADP from the cytoplasm to the mitochondria. The activation of glycolysis pathway can produce more pyruvate into the downstream pathway of mitochondria and increase the level of oxidative phosphorylation, as well as the production of ATP. ANT2 plays a very important role as a transporter in this process. ANT2 protein is closely related to glycolysis metabolism. In the case of oxygen injury, ATP can be pumped into mitochondria to maintain the function and morphology of mitochondria. In addition, ANT is the main component of mitochondrial permeability transport channel, which regulates the opening of MPTP pores and participates in apoptosis^32^. Under physiological conditions, the pore diameter of MPTP is about 0.2–0.3 μm, allowing small molecules with molecular weight less than 1.5 KD to pass through. Under normal physiological conditions, it is alternately open and closed, which is involved in regulating the pH value and charge in the mitochondrial matrix and maintaining the stability of the mitochondrial environment^33^. Many factors can affect the switch of MPTP, among which ANT located in the inner membrane of mitochondria plays an important role. We concluded that the combination of UL16 and ANT2 to regulate the level of intracellular ATP is a new mechanism of HSV-1 affecting host cell metabolism.

d-Gal is an isomer of glucose, which is converted to glucose through glucose transporters in vivo and participates in glucose metabolism. We established the model of mitochondrial dysfunction in HUVEC cells treated with d-Gal. Then we transfected UL16 into HUVEC cells treated with d-Gal and found that UL16 could restore the function of damaged mitochondria, decrease the ROS level, decrease the mitochondrial membrane potential, restore the partially destroyed mitochondrial structure, and increase the intracellular ATP production level. It also shows that the viral protein UL16 promotes the improvement of mitochondrial function.

**Figure 6:**
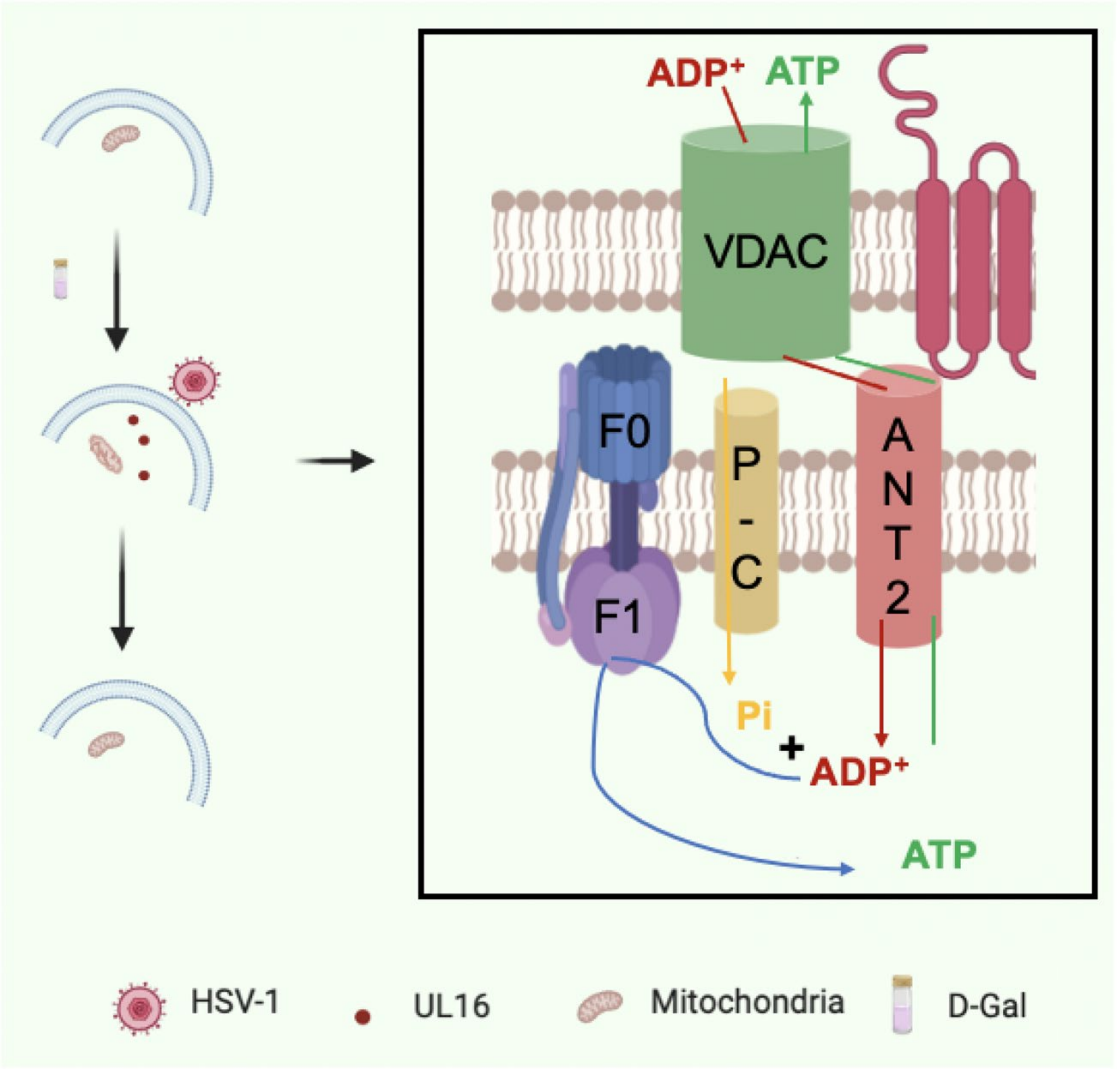
UL16 protein reveals a novel mechanism of the interaction between viral genes and host cells.

We found for the first time that herpesvirus protein UL16 directly acts on mitochondria and promotes the improvement of mitochondrial function (Fig. 6). The interaction between UL16 and ANT2 will help to understand the relationship between herpesvirus and host cell energy metabolism, which will contribute to the antiviral goal of drug design. Moreover, it has important value in the treatment of diseases such as mitochondrial dysfunction or mitochondrial diseases. However, the mechanism of UL16 regulating mitochondrial function needs further study.

## Supplementary Information

Supplementary Information 1.

## Data Availability

All relevant data are within the paper.

## References

[1] Waggoner-Fountain LA, Grossman LB (2004). Herpes simplex virus. Pediatr. Rev..

[2] El-Bacha T, Da Poian AT (2013). Virus-induced changes in mitochondrial bioenergetics as potential targets for therapy. Int. J. Biochem. Cell Biol..

[3] Mor I, Cheung EC, Vousden KH (2011). Control of glycolysis through regulation of PFK1: Old friends and recent additions. Cold Spring Harb. Symp. Quant. Biol..

[4] Abrantes JL (2012). Herpes simplex type 1 activates glycolysis through engagement of the enzyme 6-phosphofructo-1-kinase (PFK-1). Biochimica et Biophysica Acta (BBA) Mol. Basis Dis..

[5] Vastag L, Koyuncu E, Grady SL, Shenk TE, Rabinowitz JD (2011). Divergent effects of human cytomegalovirus and herpes simplex virus-1 on cellular metabolism. PLoS Pathog..

[6] Munger J, Bajad SU, Coller HA, Shenk T, Rabinowitz JD (2006). Dynamics of the cellular metabolome during human cytomegalovirus infection. PLoS Pathog..

[7] Li S (2019). Enterococcus faecalis sir2-like gene enhances aerobic metabolism of themselves and mitochondrial respiration of mammal cells to bring about improving metabolic syndrome through the PGC-1α pathway. J. Tissue Eng. Regen. Med..

[8] Fei Z (2019). Rhodotorula glutinis as a living cell liposome to deliver polypeptide drugs in vivo. Drug Deliv..

[9] Wang Y (2019). Rhodosporidium toruloides sir2-like genes remodelled the mitochondrial network to improve the phenotypes of ageing cells. Free Radic. Biol. Med..

[10] Seth RB, Sun L, Ea C-K, Chen ZJ (2005). Identification and characterization of MAVS, a mitochondrial antiviral signaling protein that activates NF-κB and IRF3. Cell.

[11] Castanier C, Garcin D, Vazquez A, Arnoult D (2010). Mitochondrial dynamics regulate the RIG-I-like receptor antiviral pathway. EMBO Rep..

[12] Munger J (2008). Systems-level metabolic flux profiling identifies fatty acid synthesis as a target for antiviral therapy. Nat. Biotechnol..

[13] Wagner BK (2008). Large-scale chemical dissection of mitochondrial function. Nat. Biotechnol..

[14] Postigo A, Ferrer PE (2009). Viral inhibitors reveal overlapping themes in regulation of cell death and innate immunity. Microb. Infect..

[15] Siu GKY (2016). Hepatitis C virus NS5A protein cooperates with phosphatidylinositol 4-kinase IIIα to induce mitochondrial fragmentation. Sci. Rep..

[16] Tanaka M, Sata T, Kawaguchi Y (2008). The product of the herpes simplex virus 1 UL7 gene interacts with a mitochondrial protein, adenine nucleotide translocator 2. Virol. J..

[17] Chadha P (2017). Domain interaction studies of herpes simplex virus 1 tegument protein UL16 reveal its interaction with mitochondria. J. Virol..

[18] Derakhshan M, Willcocks MM, Salako MA, Kass GEN, Carter MJ (2006). Human herpesvirus 1 protein US3 induces an inhibition of mitochondrial electron transport. J. Gen. Virol..

[19] Corcoran JA, Saffran HA, Duguay BA, Smiley JR (2009). Herpes simplex virus UL12.5 targets mitochondria through a mitochondrial localization sequence proximal to the N terminus. J. Virol..

[20] Hackenberg H, Klingenberg M (1980). Molecular weight and hydrodynamic parameters of the adenosine 5'-diphosphate-adenosine 5'-triphosphate carrier in triton X-100. Biochemistry.

[21] Ding Q (2015). HIV-1 coreceptor CXCR4 antagonists promote clonal expansion of viral epitope-specific CD8+ T cells during acute SIV infection in rhesus monkeys in vivo. J. Acquir. Immune Defic. Syndr..

[22] Zhou C, Cunningham L, Marcus AI, Li Y, Kahn RA (2006). Arl2 and Arl3 regulate different microtubule-dependent processes. Mol. Biol. Cell.

[23] Aerts S (2006). Gene prioritization through genomic data fusion. Nat. Biotechnol..

[24] Li Z (2013). Overexpression of malic enzyme (ME) of *Mucor circinelloides* improved lipid accumulation in engineered *Rhodotorula glutinis*. Appl. Microbiol. Biotechnol..

[25] Petiot E (2010). Kinetic characterization of vero cell metabolism in a serum-free batch culture process. Biotechnol. Bioeng..

[26] Chen J, Aronow BJ, Jegga AG (2009). Disease candidate gene identification and prioritization using protein interaction networks. BMC Bioinform..

[27] Zhang C, Jiang H, Wang P, Liu H, Sun X (2017). Transcription factor NF-kappa B represses ANT1 transcription and leads to mitochondrial dysfunctions. Sci. Rep..

[28] Kaarbø M (2011). Human cytomegalovirus infection increases mitochondrial biogenesis. Mitochondrion.

[29] Portman MA (2000). Adenine nucleotide translocator in heart. Mol. Genet. Metab..

[30] Gouriou Y, Alam MR, Harhous Z, Silva C, Bidaux G (2020). ANT2-mediated ATP import into mitochondria protects against hypoxia lethal injury. Cells.

[31] Chevrollier A, Loiseau D, Reynier P, Stepien G (2011). Adenine nucleotide translocase 2 is a key mitochondrial protein in cancer metabolism. BBA—Bioenerg..

[32] Bernardi P, Di Lisa F (2015). The mitochondrial permeability transition pore: Molecular nature and role as a target in cardioprotection. J. Mol. Cell Cardiol..

[33] Bauer MKA, Schubert A, Rocks O, Grimm S (1999). Adenine nucleotide translocase-1, a component of the permeability transition pore, can dominantly induce apoptosis. J. Cell Biol..

