# Supplementary material for: The UL16 protein of HSV-1 promotes the metabolism of cell mitochondria by binding to ANT2 protein
Source: Sci Rep. 2021 Jul 7;11:14001. doi: 10.1038/s41598-021-93430-2 (PMC8263751; doi:10.1038/s41598-021-93430-2)

**Supplemental material for**

**The UL16 protein of HSV-1 promotes the metabolism of cell mitochondria by binding to ANT2 protein.**

Shiyu Li^1,^ ^2#^, Shuting Liu^1#^, Zhenning Dai^3^, Qian Zhang^1^, Yichao Xu^2^, Youyu Chen^1^, Zhenyou Jiang^4*^, Wenhua Huang^2*^, Hanxiao Sun^1*^

^1^Institute of Genomic Medicine, College of Pharmacy, Jinan University, Guangzhou, 510632, China.

^2^Department of Anatomy, School of Basic Medical Sciences, Southern Medical University, Guangzhou, 510515, China.

^3^Department of Stomatology, Guangdong Second Traditional Chinese Medicine Hospital, Guangzhou, 510095, China.

^4^Departments of Microbiology and Immunology, Jinan University, Guangzhou, 510632, China.

**^#^**These authors contributed equally to this work.

^*^Corresponding author:

**Zhenyou Jiang,**

E-mail: tjzhy1998@163.com

**Wenhua Huang**,

E-mail: huangwenhua2009@sina.com

**Hanxiao Sun**,

E-mail: hanxiaosun718@jnu.edu.cn

**Fig. S1 Western Blot**


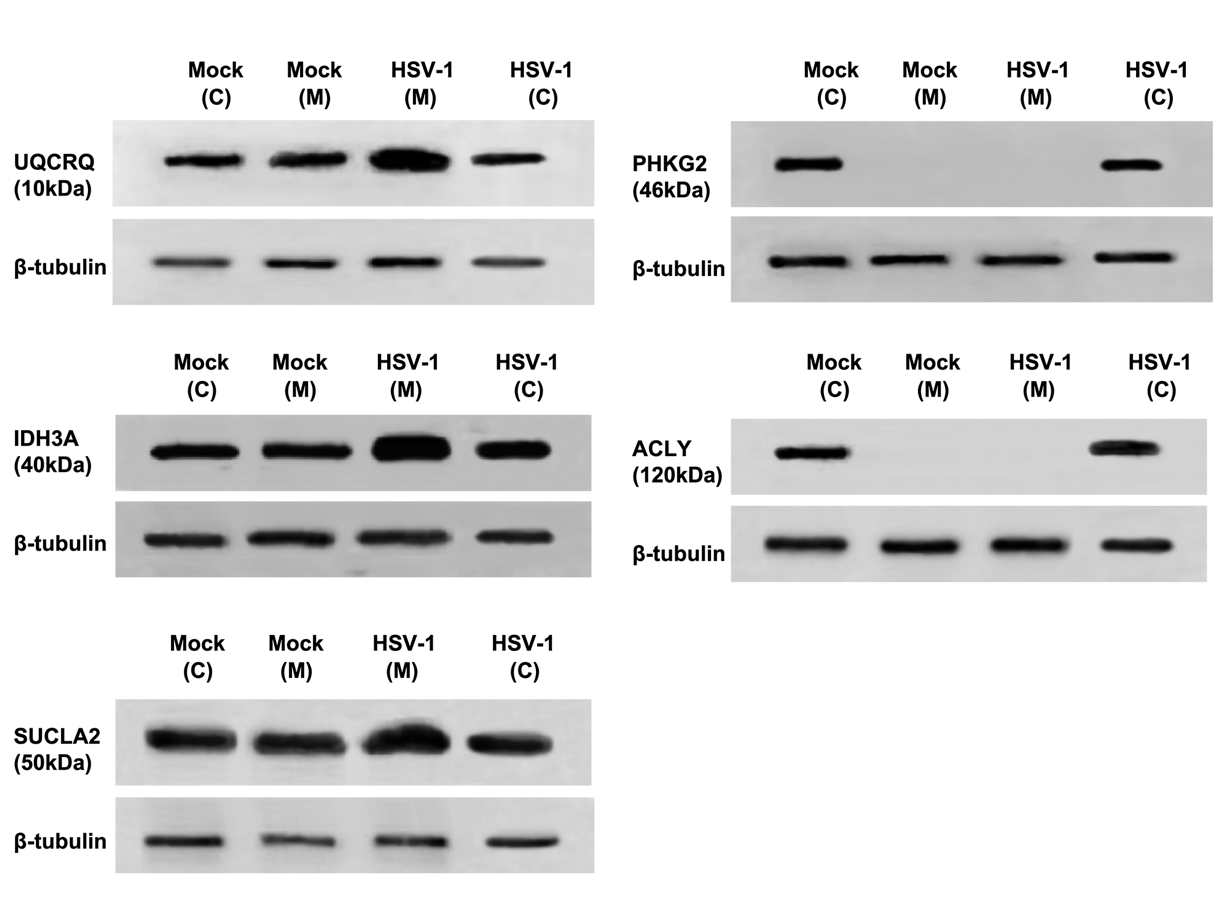


**Fig. S2 Mass spectrometric analysis of viral protein UL16 bound to ANT2**


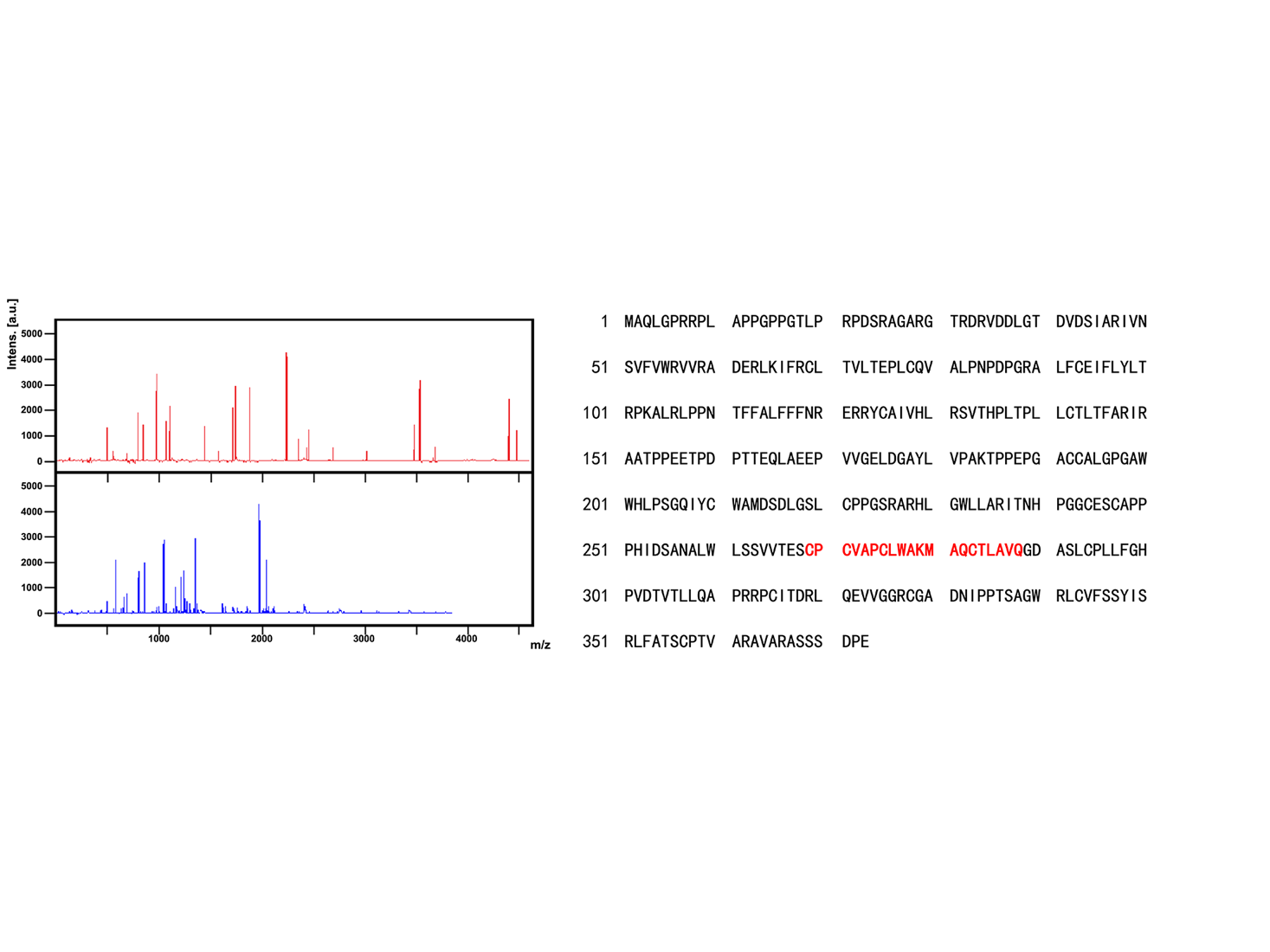


**Table S1. Primer of candidate genes in human**

| **Gene** | **Forward primer (5'-3')** | **Reverse primer (5'-3')** |
| --- | --- | --- |
| ACLY | TCGGCCAAGGCAATITCAGAG | CGAGCATACTTGAACCGATTCT |
| SLC25A4 | GCTTTGCGTCCTTCATCTT | ACCTCCCTCTGCTTCGTCT |
| PHKG2 | GTTCCTGGTGTTTGACCTGA | GATAAAGCTCACTGCCTCCA |
| PFKFB3 | CATTTGGAAACTCGGCAGCA | AGGATCTCAGGGCTCACAGCA |
| IDH1 | ACGGTCTTCAGAGAAGC | GGTGTAGATACCAAAAGATAAGAAT |
| IDH3A | AAATGAATTCGGGTCCGCGTG | GGGGAAGCTTGTCTTTGACTCTACGA |
| ACO2 | CCGCAGAACCAGATTCCCTC | AATTTCCCTGTTTAGCACCGTT |
| SUCLA2 | CATGCTGCTCCATGCTTCAT | CATGCTCATGACATACAAACA |
| UQCRQ | CGCACGTCTTCACTAAAGG | AAACACTACAAACTGCGGC |
| ANT2 | TTTGAACATGACGGATGCCGC | CCTGCACTGACACATTAAAGC |

**Table S2. Functions of candidate genes in human**

|  |  | **Description ^a^** | | |
| --- | --- | --- | --- | --- |
| **Gene ID** | **Symbol** | **Biological process** | **Cellular component** | **Molecular function** |
| 47 | ACLY | citrate metabolic process | mitochondrion | ATP binding |
| 292 | SLC25A5 | Energy reserve metabolic process | positive regulation of cell proliferation | protein binding |
| 3419 | IDH3A | carbohydrate metabolic process | mitochondrial matrix | NAD binding |
| 5261 | PHKG2 | carbohydrate metabolic process | phosphorylase kinase complex | ATP binding |
| 27089 | UQCRQ | cellular metabolic process | mitochondrial inner membrane | ubiquinol-cytochrome-c reductase activity |
| 407183 | PFKFB3 | fructose metabolic process | cytoplasm | [ATP binding](http://amigo.geneontology.org/amigo/term/GO:0005524) |
| 39590764 | ANT2 | ADP, ATP carrier protein 2 | mitochondrial intermembrane space | ADP, ATP carrier protein 2 |

Gene products have several functions, which have many GO terms, but only one is included in the table.

^a^ Biological process, cellular component, and molecular functions predicted by the GO annotation system and NCBI database.

Western Blot

Fig.1E

SLC25A4


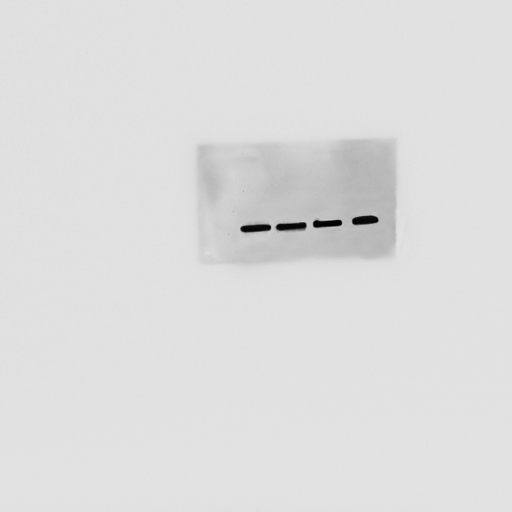

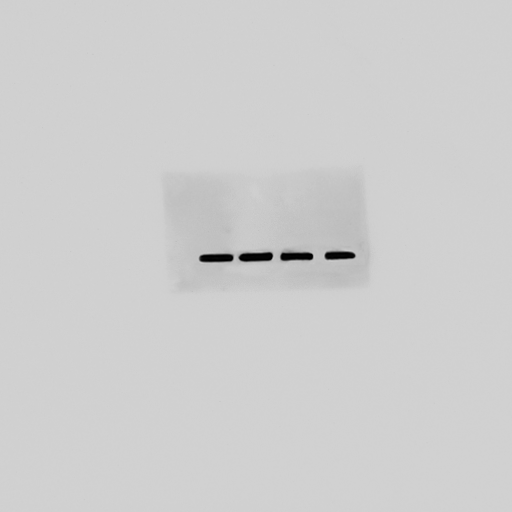


PFKFB3


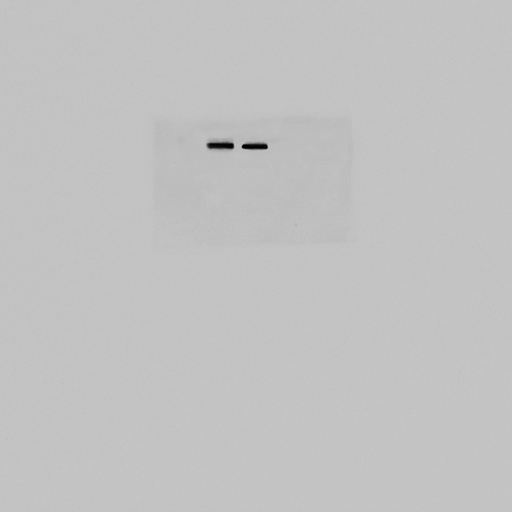

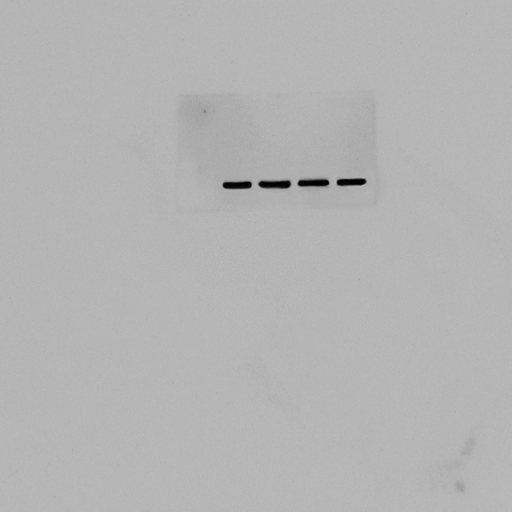


ACO2


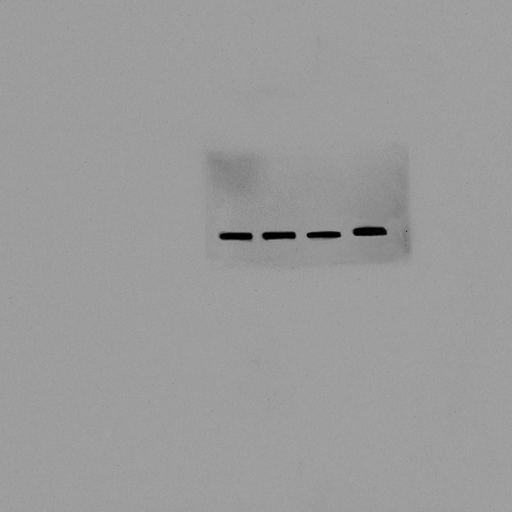

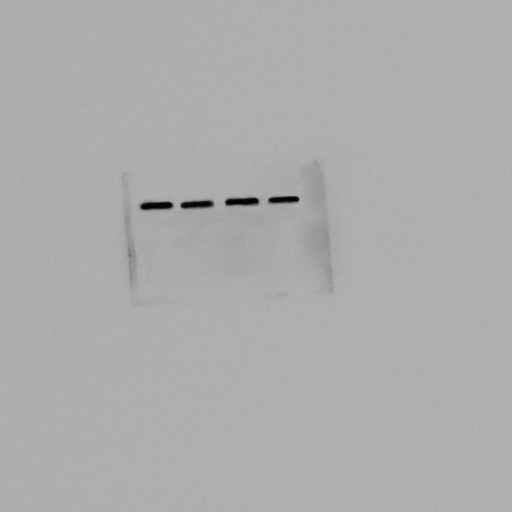


ANT2 + UL16


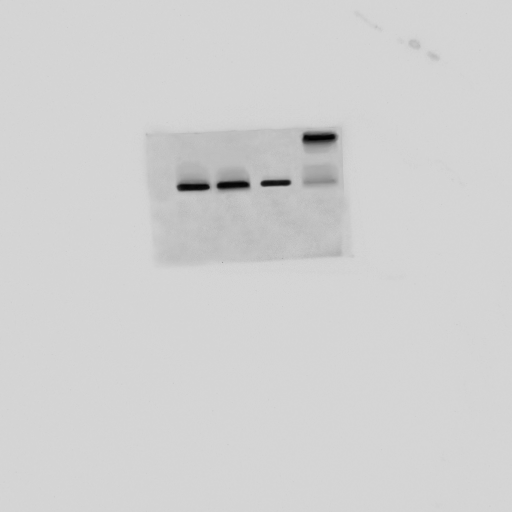

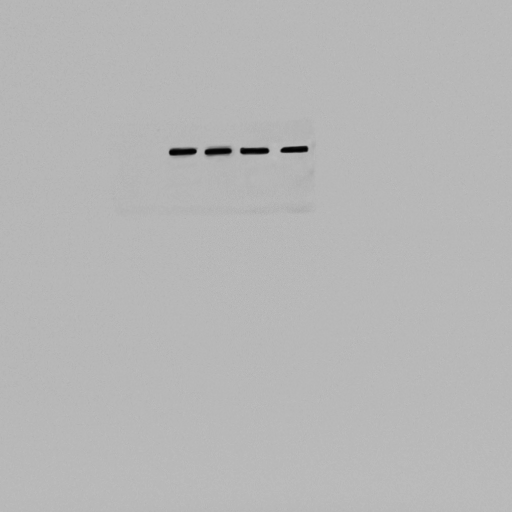


IDH1


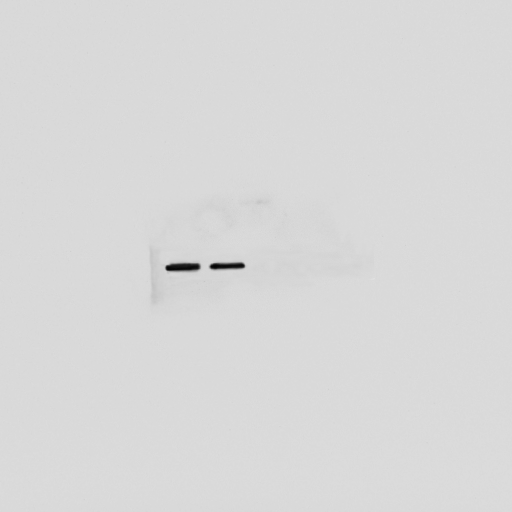

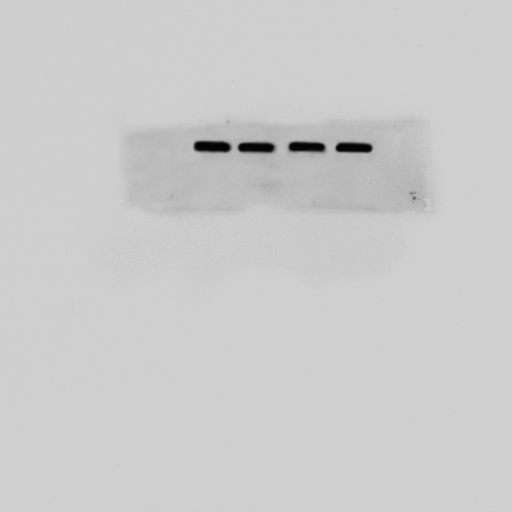


Fig.2A


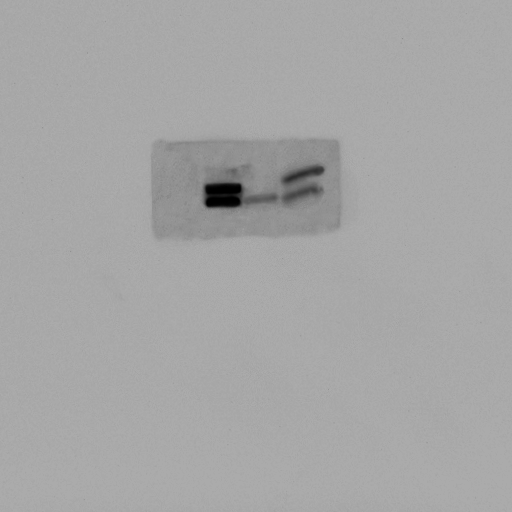


Fig.2B


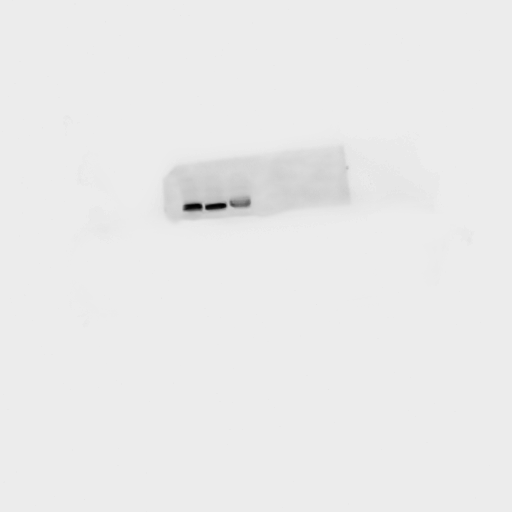

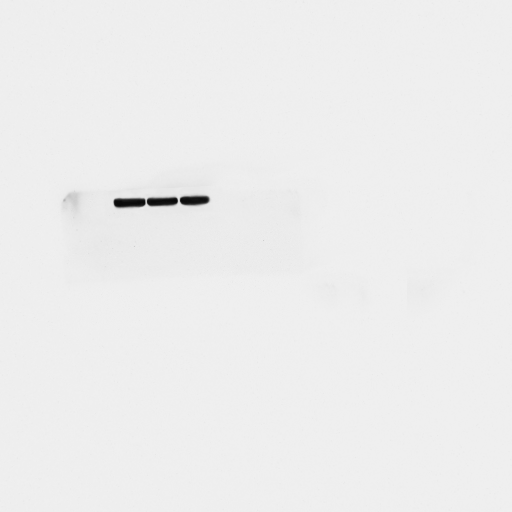


Fig.4B


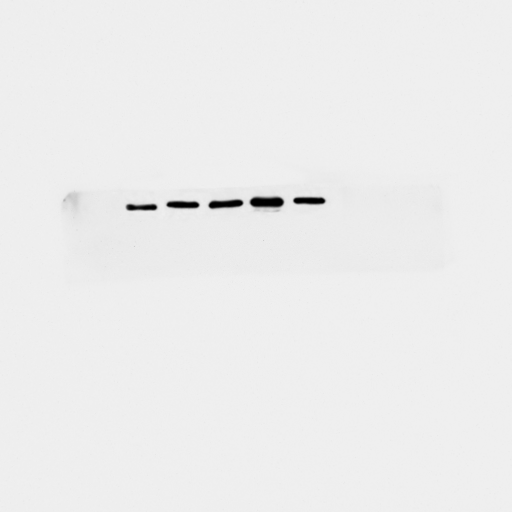

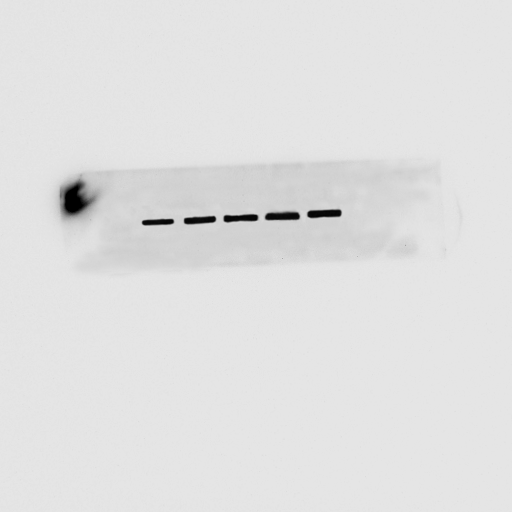

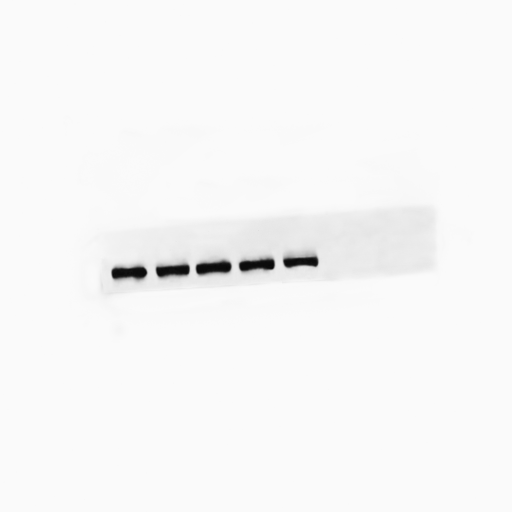


Fig.5A


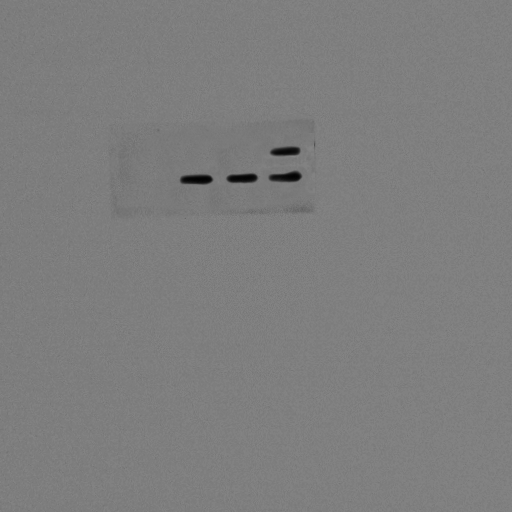


Fig.S1

UQCRQ


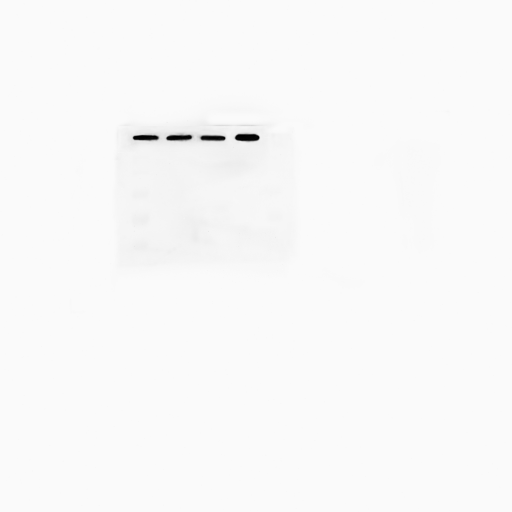

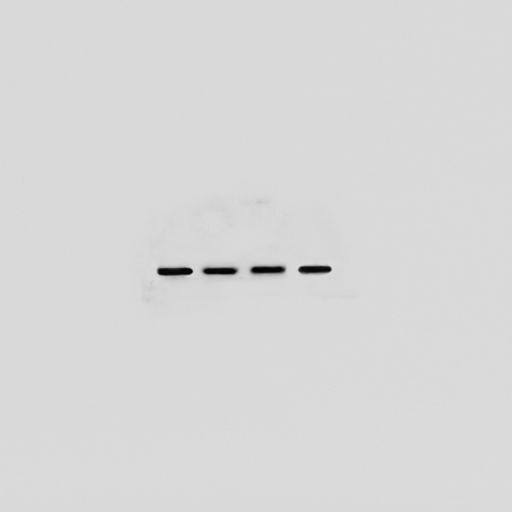


PHKG2


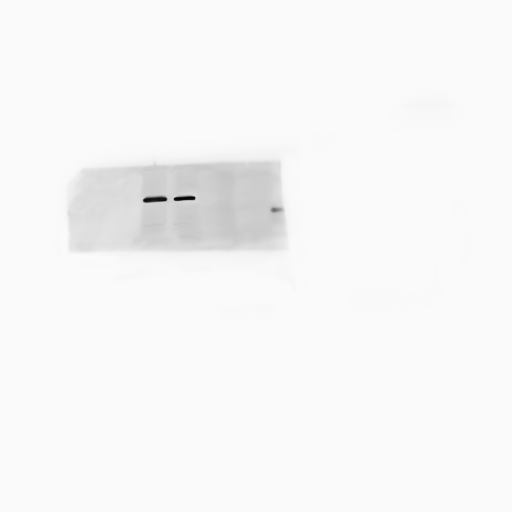

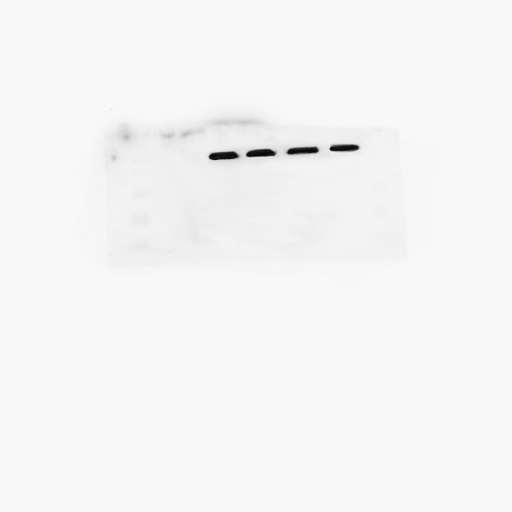


IDH3A


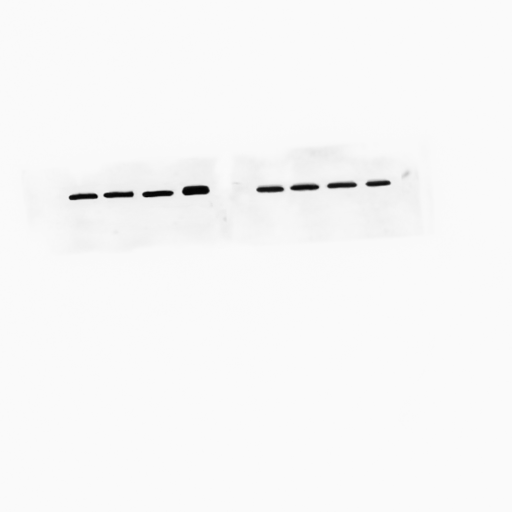


ACLY


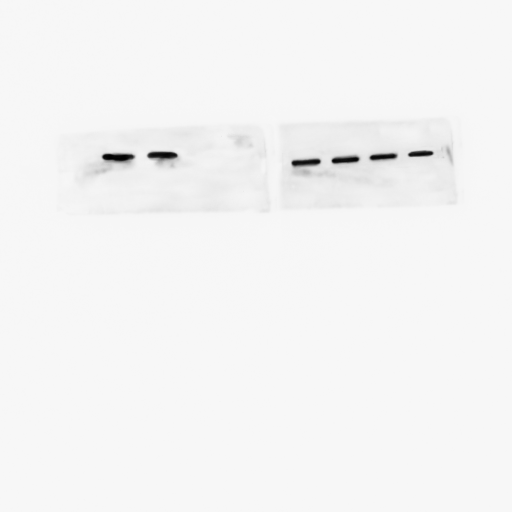


SUCLA2


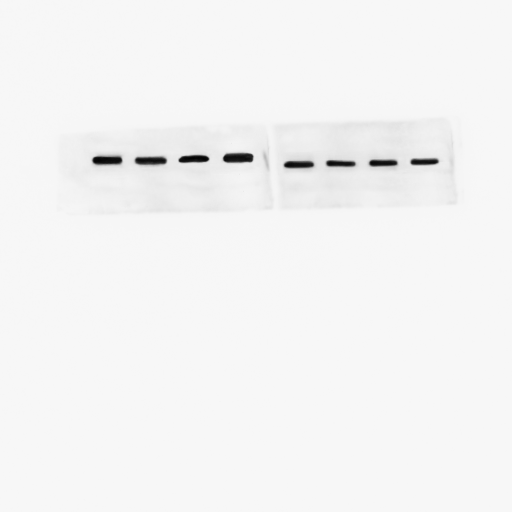

Supplement: Supplementary file 1 — Supplementary Information 1. [file 41598_2021_93430_MOESM1_ESM.docx]
